# Mitochondrial Tim9 protects Tim10 from degradation by the protease Yme1

**DOI:** 10.1042/BSR20150038

**Published:** 2015-05-19

**Authors:** Michael P. Spiller, Liang Guo, Qi Wang, Peter Tran, Hui Lu

**Affiliations:** *Manchester Institute of Biotechnology, Faculty of Life Sciences, University of Manchester, 131 Princess Street, Manchester M1 7DN, U.K.

**Keywords:** adenosine 5′-triphosphate (ATP)-independent chaperone, disulfide bond, protein stability, protein–protein interaction, small Tim protein, AAA, ATPases associated with diverse cellular activities, AAC, ATP/ADP carrier protein, IM, inner membrane, IMS, intermembrane space, IP, immunoprecipitation, MIA, mitochondrial import and assembly, OM, outer membrane, RT, room temperature, TCA, trichloroacetic acid, Tim, translocase of inner membrane, WT, wild-type, YPD, yeast extract protein dextrose

## Abstract

Translocase of IM (inner membrane; Tim)9 and Tim10 are essential homologue proteins of the mitochondrial intermembrane space (IMS) and form a stable hexameric Tim9–Tim10 complex there. Redox-switch of the four conserved cysteine residues plays a key role during the biogenesis of these proteins and, in turn, the Tim proteins play a vital chaperone-like role during import of mitochondrial membrane proteins. However, the functional mechanism of the small Tim chaperones is far from solved and it is unclear whether the individual proteins play specific roles or the complex functions as a single unit. In the present study, we examined the requirement and role for the individual disulfide bonds of Tim9 on cell viability, complex formation and stability using yeast genetic, biochemical and biophysical methods. Loss of the Tim9 inner disulfide bond led to a temperature-sensitive phenotype and degradation of both Tim9 and Tim10. The growth phenotype could be suppressed by deletion of the mitochondrial i-AAA (ATPases associated with diverse cellular activities) protease Yme1, and this correlates strongly with stabilization of the Tim10 protein regardless of Tim9 levels. Formation of both disulfide bonds is not essential for Tim9 function, but it can facilitate the formation and improve the stability of the hexameric Tim9–Tim10 complex. Furthermore, our results suggest that the primary function of Tim9 is to protect Tim10 from degradation by Yme1 via assembly into the Tim9–Tim10 complex. We propose that Tim10, rather than the hexameric Tim9–Tim10 complex, is the functional form of these proteins.

## Abstract 2

Tim9 and Tim10 are essential homologue proteins forming a stable complex in mitochondria. The present study suggests that primary function of Tim9 is to protect Tim10 from degradation by Yme1 via assembly into the Tim9–Tim10 complex.

## INTRODUCTION

Translocase of IM (inner membrane; Tim)9 and Tim10 are essential members of the ‘small Tim’ (also known as ‘Tiny Tim’) protein family of the mitochondrial intermembrane space (IMS). They act as ATP-independent chaperones for precursors destined for the IM and outer membrane (OM) of mitochondria, guiding them from the mitochondrial pore complex (GIP–TOM, general import pore–translocase of the outer membrane; complex) across the aqueous environment of the IMS to their destination [1,2]. In the IMS, the small Tim proteins form into hexameric 70k Da α_3_β_3_ complexes, e.g. the essential Tim9–Tim10 complex or the non-essential Tim8–Tim13 complex. These hexameric complexes are considered to be the functional forms of these proteins [3–5].

**Figure 1:**
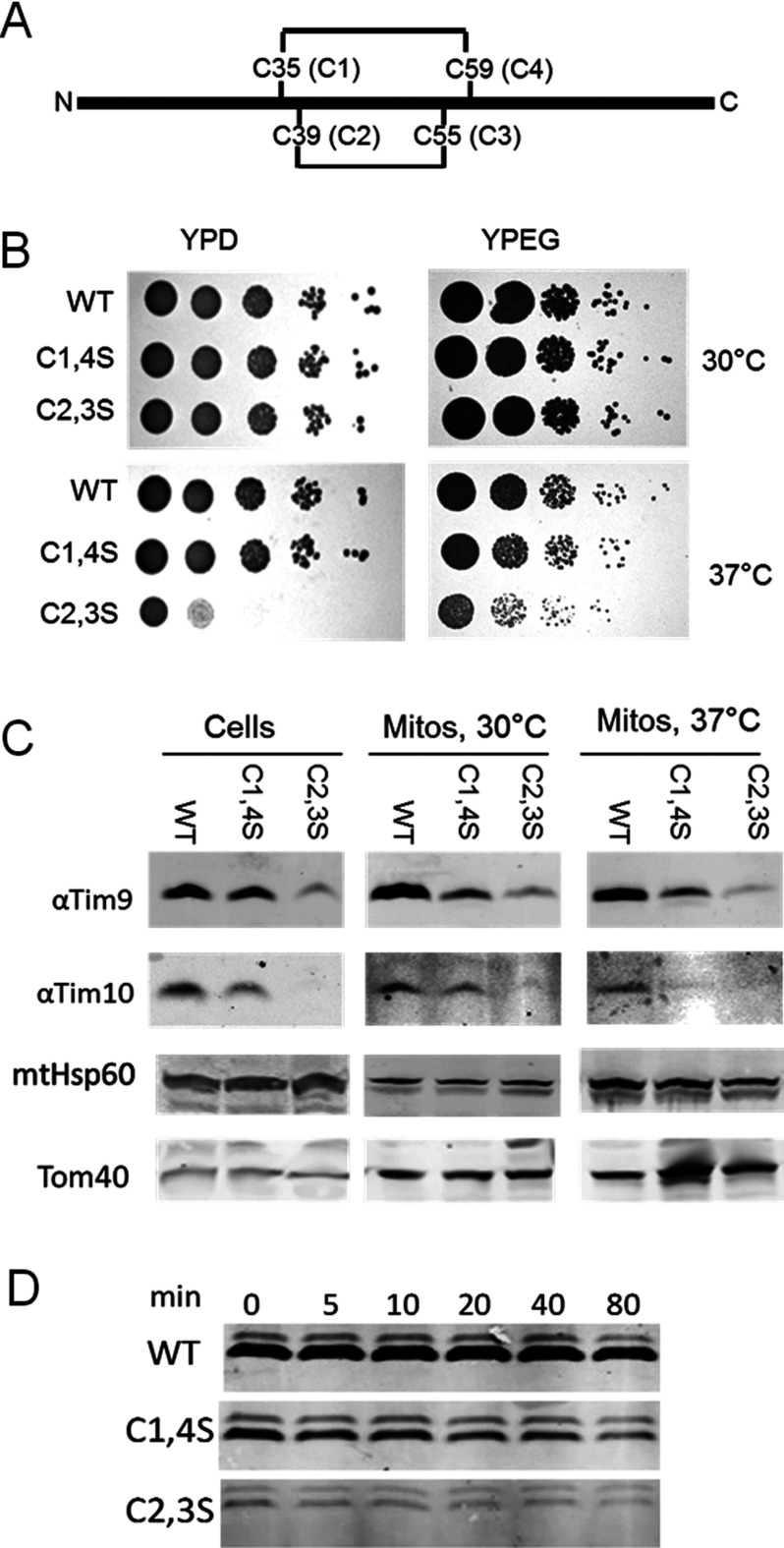
Mutation of Tim9 cysteine residues causes a growth defect and small Tim instability (**A**) Schematic representation of the primary structure of Tim9 with its two disulfide bonds and the conserved cysteine motifs. (**B**) Spot tests for growth of *tim9* cysteine mutants on fermentable (YPD) and non-fermentable (YPEG) carbon sources at 30°C and 37°C (**C**) Levels of mitochondrial proteins from the WT and *tim9* mutant cells and mitochondria. (**D**) Time course of mitochondrial Tim9 levels following shift to 37°C. Mitochondria were lysed at the indicated time points following temperature shift and Tim9 was detected by Western blotting.

All the small Tim proteins are synthesized in the cytoplasm and are characterized by the presence of four conserved cysteine residues, arranged in two CX_3_C motifs [2]. These strictly conserved cysteine residues are believed to be required for the import of small Tim precursors into the IMS, as well as the folding and stability of the mature proteins [6–10]. In mitochondria, these cysteine residues form two pairs of intramolecular disulfide bonds which connect the two halves of the protein. For example, in Tim9, Cys^35^ (C1) is linked to Cys^59^ (C4) and Cys^39^ (C2) to Cys^55^ (C3) (Figure 1A). Whereas only cysteine-reduced, unfolded precursor proteins can be imported across the mitochondrial OM into the IMS [9,11,12], formation of these disulfide bonds is important for the folding and retention of the small Tim proteins in the mitochondrion [9,11,13,14]. This so-called ‘folding-trap’ import mechanism is mediated by the MIA (mitochondrial import and assembly) pathway, in which reduced small Tim proteins are oxidized by the oxidoreductase Mia40, which is then recycled back to its oxidized state by the FAD-dependent oxidase Erv1 (essential for respiration and viability). The oxidation reaction occurs through the formation of a covalently-bonded intermediate between the second cysteine residue of the Mia40 CPC motif and a cysteine residue in the substrate precursor (e.g., the small Tim proteins, Cox17 (Cytochrome C OXidase) family). The interaction of Mia40 with precursor proteins is a two-step process, initiated by non-covalent hydrophobic interaction and followed by covalent disulfide bond formation between Mia40 and its substrate, which lead to oxidative folding of the substrate in the IMS [6,15–17]. Consequently, the oxidized small Tim proteins interact with their partner proteins, forming the stable hexameric α_3_β_3_ complexes in the IMS. For the Tim9–Tim10 complex, the assembly process is driven firstly by electrostatic protein–protein interactions and followed by packing of hydrophobic interactions between individual subunits [18]. Kinetic studies revealed the presence of tetrameric assembly intermediates prior to formation of the hexameric Tim9–Tim10 complex [19,20].

Recently, there has been some doubt cast on the relative importance of oxidative folding for import of the IMS proteins as Weckbecker et al. [21] showed that the IMS protein Atp23 (ATP synthase) could be imported into mitochondria *in vitro* despite the removal of all of its cysteine residues. Furthermore, Mia40 has been implicated in cysteine-independent import of IM protein Tim22 [22] and in the oxidation-independent import of Ccs1 (the copper chaperone for Sod1 (SuperOxide Dismutase)) [23]. For Tim9, import of radiolabelled precursor into purified mitochondria (so-called *in organelle* methods) identified an interaction between Mia40 and the first cysteine (Cys^35^) of Tim9 as critical to Tim9 folding and imports [14]. However, work by Baker et al. [24] showed that mutation of this cysteine is not deleterious for yeast viability *in vivo*, suggesting that there does not appear to be a specific requirement for any particular cysteine residue for the import and function of the small Tim proteins. Furthermore, the work of Baker et al. [24] identified that loss of cysteine residues in IMS proteins leads to degradation by the IM AAA (ATPases associated with diverse cellular activities) protease Yme1 (yeast mitochondrial escape), which probably acts to eliminate mis-folded proteins in the IMS. Therefore, it may be that import might simply require non-covalent hydrophobic interaction with Mia40 and the oxidoreductase function of Mia40 is to further ensure the production of stable and functional proteins.

The work described above supported earlier studies [25–27] that suggested the small Tim proteins co-stabilize each other *in vivo*. In terms of the function of Tim9 and Tim10, it is unclear whether the individual subunits play specific roles [28] or whether the hexamer functions as a single unit. It has been suggested that Tim10 is primarily involved in client binding (possibly in the monomer form), whereas Tim9 stabilizes Tim10 in the Tim9–Tim10 complex. This is based on the observations that Tim9 does not interact with the transmembrane regions of the Tim9–Tim10 client AAC (ATP/ADP carrier protein) as detected by NMR and does not bind at all to AAC in peptide spot assays [29]. Moreover, in comparison with Tim10, Tim9 is more difficult to cross-link to AAC, although it can be detected following modification of Tim9 sequence [30]. Meanwhile, Tim10 can be readily cross-linked to AAC under a variety of conditions [31,32] and interacts with several regions of AAC by peptide spot assay. This binding was severely disrupted by loss of the Tim10 N-terminus [29] and the AAC-binding property could be transferred to the Tim10 homologue Tim13 by adding the Tim10 N-terminus [33], indicating a critical role for this part of Tim10 in client interaction. Moreover, mutants in which the Tim9–Tim10 hexamer is not detectable by blue-native PAGE [27,28], but still support cell growth, suggest that the small Tim proteins can function as individual units to chaperone clients. Taken together, recent developments suggest a model in which oxidative folding, rather than being essential for import, is important for assembly of the Tim9–Tim10 complex, which in turn might protect the individual subunits from degradation.

In the present study, we tested the requirements for the inner and outer disulfide bonds of Tim9 as well as the effects of *yme1* deletion on cell growth, Tim9–Tim10 complex formation and protein stability, using various yeast genetic and protein characterization methods. First, in line with the work of Baker et al. [24] we found that loss of the Tim9 inner disulfide bond led to a temperature-sensitive phenotype. This temperature sensitivity could be suppressed by deletion of the Yme1 protease. Furthermore, we identified that a specific feature of this rescue was a restoration of the levels of Tim10, regardless of Tim9 levels, suggesting that the critical factor in small Tim function is the Tim10 itself. Then, we investigated the effect of Tim9 cysteine mutants on Tim9–Tim10 formation *in vitro* using purified proteins. Our results showed that both mutant Tim9 proteins were defective in hexameric Tim9–Tim10 complex formation, including the outer disulfide bond mutant (Tim9C1,4S) that supports growth at both 30°C and 37°C. We found that this mutant (Tim9C1,4S) appears to form a complex, but of a smaller size, whereas the temperature-sensitive inner disulfide bond mutant (Tim9C2,3S) was predominantly unassembled. Finally, we examined the thermal stability and proteinase resistance of the wild-type (WT) and mutant complexes. Taken together, our results suggest that the primary function of Tim9 forming a complex with Tim10 is to stabilize the Tim10 from degradation by Yme1 and that probably Tim10, rather than the hexameric Tim9–Tim10 complex, is the functional moiety of these proteins.

## EXPERIMENTAL

### Site-directed mutagenesis

Primers used in the present study are summarized in Supplementary Table S1. A construct encoding full-length, WT Tim9 including native promoter and terminator sequences cloned into the yeast *URA3* shuttle vector pRS416 (pLG025) was used as a DNA template to generate cysteine mutants of Tim9. The primers 9C1F and 9C1R used to mutate Cys^35^ (C1) to serine and the primers 9C2F and 9C2R were used to mutate Cys^39^ (C2) to serine. These single cysteine mutant plasmids were then used as templates for the mutant of Cys^55^ (C3) and Cys^59^ (C4) respectively. C3 was mutated to serine using the primers 9C3F and 9C3R and C4 was mutated to serine using 9C4F and 9C4R. The result was the generation of a Tim9C1,4S mutant (pLG030) and Tim9C2,3S (pLG031). The WT and mutant *tim9* genes were then cloned into the *LEU2* vector pRS315 to generate pLG040 (Tim9 WT), pLG042 (Tim9C1,4S) and pLG041 (Tim9C2,3S).

### Generation of yeast strains

A *tim9Δ* strain was generated by the transformation of pRS416–Tim9 (pLG025) into the WT strain BY4742. This strain was then used for deletion of the genomic *TIM9* gene by homologous recombination. The phleomycin (*ble*) gene was amplified from the plasmid [pUG66] using the primers 9loxF and 9loxR containing regions homologous to the upstream and downstream sequences of *TIM9*. This PCR product was then transformed into BY4742 + pLG025 and transformants were selected on 100 μg/ml phleomycin. Recombination into the *TIM9* locus was confirmed by PCR and to distinguish between recombination into the genomic *TIM9* locus as opposed to the plasmid copy, transformants were streaked on to 5-fluoroorotic acid (5-FOA), with those with the genomic copy knocked out requiring the presence of the *URA3* plasmid and therefore unable to survive on 5-FOA. This strain (MPS4) was then used for a further round of PCR-based homologous recombination to knockout the *YME1* gene. This PCR used the plasmid pFa6a–KanMX4 as template to amplify the *KanMX4* gene that confers G418 resistance using the primers Yme1F1 and Yme1R1. The PCR product was transformed into the MPS4 strain with the *tim9* gene knocked out and transformants were selected on 200 μg/ml G418 and confirmed by PCR to generate strain MPS19. To generate the *tim9* mutant strains in the in the *YME1* and *yme1Δ* backgrounds, the WT and mutant *TIM9 LEU2* plasmids pLG040, pLG041 or pLG042 were transformed into MPS4 and MPS19 respectively. WT *TIM9 URA3* plasmid was removed by streaking on 5-FOA to generate MPS21 (*tim9C1,4S*), MPS22 (*tim9C2,3S*), MPS28 (*tim9C1,4S yme1Δ*) and MPS29 (*tim9C2,3S yme1Δ*).

### Cell viability assay

Selected individual colonies were grown YPD (yeast extract protein dextrose) overnight before spot-testing on YPD or YPEG (yeast extract peptone ethanol glycerol) plates at 30°C or 37°C.

### Steady-state mitochondrial protein analysis

Mitochondrial preparations were performed as per [34,35]. For analysis by Western blotting, mitochondria were pelleted and resuspended in 1× SDS sample buffer + DTT. Total cell extracts were performed as in [36].

### Pulse chase analysis

Cells were grown in synthetic selective media lacking methionine overnight, then diluted to pre-log phase in fresh media and grown for 6 h. Cells were pelleted at 3000 g and resuspended in 500 μl of medium and incubated at 30°C for 5 min in a shaking waterbath. Pulse-labelling was performed by the addition of 5μl (50 μCi) of 35S Express Protein labeling mix (Perkin–Elmer) followed by a further 5 min incubation. Chase was initiated by addition of unlabelled methionine to 20 mM and 10 μg/ml cycloheximide. Samples were taken at regular time points by freezing cells in 10% TCA (trichloroacetic acid). For immunoprecipitations (IPs), cells were thawed and pelleted, then resuspended in urea lysis buffer (8M urea, 2% SDS, 50 mM Tris/HCl, pH 7.5) and broken by glass bead lysis. Proteins were denatured by heating at 95°C for 5 min and diluted in 1ml of IP buffer (150 mM NaCl, 62.5 mM Tris, pH 8, 1.25 Triton-X100, 5 mM EDTA). Samples were cleared by centrifugation at 14000 g for 40 min at 4°C. Antibody was added at 1 μl/unit 600 of cells and incubated for 1 h at room temperature (RT), followed by addition of Protein A sepharose and incubation for 1 h. Antibody was denatured by the addition of 1× SDS sample buffer + DTT and heating at 95°C for 5 min. Proteins were separated by SDS/PAGE and visualized by autoradiography and images were captured using a Typhoon phosporimager and quantified using AIDA densitometry software.

### Protein purification

All the WT and mutant small Tim proteins were expressed in *Escherichia coli* Rosetta-gami cells as a GST–Tim fusion protein (with pGEX-4T plasmid) and purified using GST affinity-binding beads followed by thrombin cleavage to release the proteins as described previously [18]. The proteins were further purified by FPLC gel-filtration chromatography (Superdex 75) using buffer AE (50 mM Tris/HCl buffer, pH 7.4, 150 mM NaCl and 1 mM EDTA) at 4°C, before further analysis.

### CD

CD analysis was performed using a JASCO J810 spectropolarimeter with a 1-mm path length quartz cuvette as described previously [37]. Each spectrum represents an average of four scans with the spectra for buffer alone subtracted. Thermal denaturation was measured at 222 nm at 1°C intervals over 5°C–90°C, with a temperature increase at 1°C/min, as described previously [38].

### Trypsin digestion

Trypsin digestion with the WT and mutant small Tim proteins were carried out at RT (approximately 20°C) from 0 to 30 min and the reactions were stopped by addition of 10× SBTI (Soybean Trypsin Inhibitor) and on ice to inhibit the reactions. The samples were analysed using 16% Tris/Tricine SDS/PAGE followed by Coomassie staining or western blotting analyses.

## RESULTS

### Tim9 cysteine mutants are viable *in vivo*

To test whether Tim9 lacking either inner or outer disulfide bonds (Figure 1A) could support cell growth, we constructed strains in which Tim9 on a low copy plasmid carrying either *tim9C1,4S* (Cys35,59Ser) or *tim9C2,3S* (Cys39,55Ser) double cysteine to serine mutations. We tested the growth of these strains by spot testing at various temperatures (Figure 1B). Our results showed that the *tim9C2,3S* mutation, which is only capable of forming the outer disulfide bond of Tim9, has significantly reduced growth at 37°C. On the other hand, the *tim9C1,4S* mutant showed no obvious growth defect, but grew slightly slower at 37°C. We also observed slow growth of *tim9C2,3S* in liquid culture at 30°C (result not shown), which makes our results generally in agreement with the work of Baker et al. [24], who went on to show that that the IM protease Yme1 can degrade mis-folded small Tim proteins. This led us to ask the question of whether the *tim9C2,3S* growth defect was caused by low levels of Tim9 and therefore might be rescued by deletion of the Yme1 protease.

### Tim9 cysteine mutant strains have reduced levels of both Tim9 and Tim10

We investigated the level of Tim9 and Tim10 in the two *tim9* disulfide mutant strains and found, as expected that both *tim9C1,4S* and *tim9C2,3S* strains had reduced levels of Tim9 and Tim10, as detected by Western blotting of total cell extracts grown at 30°C, as well as mitochondria purified at 30°C and 37°C (Figure 1C). We observed that the levels of Tim9C2,3S were lower than the levels of Tim9C1,4S and this correlated with lower levels of Tim10, especially in the *tim9C2,3S* strain. These markedly decreased levels of Tim9 and Tim10 in the *tim9C2,3S* strain provide an explanation for why it, but not the *tim9C1,4S* strain, shows a temperature-sensitive phenotype, probably due to the proteins decreasing below a critical level in *tim9C2,3S* strain. The decreased protein level in the mutant strains also suggests that the proteins are getting rapidly degraded. To investigate whether the Tim9C2,3S mutant was less stable in mitochondria compared with the WT and Tim9C1,4S mutant, we purified mitochondria from strains grown at 30°C and incubated them at 37°C. We monitored Tim9 levels after incubation of the mitochondria at 37°C for various time points by western blotting. As observed in Figure 1(C), the initial levels of Tim9C1,4S were significantly lower than those of WT Tim9, whereas the levels of Tim9C2,3S were lower still (Figure 1D, time 0). Moreover, both Tim9 mutants were similarly unstable over the time course at 37°C (Figure 1D), with the levels of Tim9 mutants dropping to approximately 50% that of the zero time point in approximately 40–80 min. Therefore, within purified mitochondria incubated at 37°C, both mutant proteins are unstable compared with the WT Tim9, but the level of Tim9C2,3S was more significantly reduced compared with Tim9C1,4S prior to mitochondria incubation at 37°C.

### *YME1* deletion rescues temperature sensitivity of the *tim9C2,3S* mutant and stabilizes Tim10

**Figure 2:**
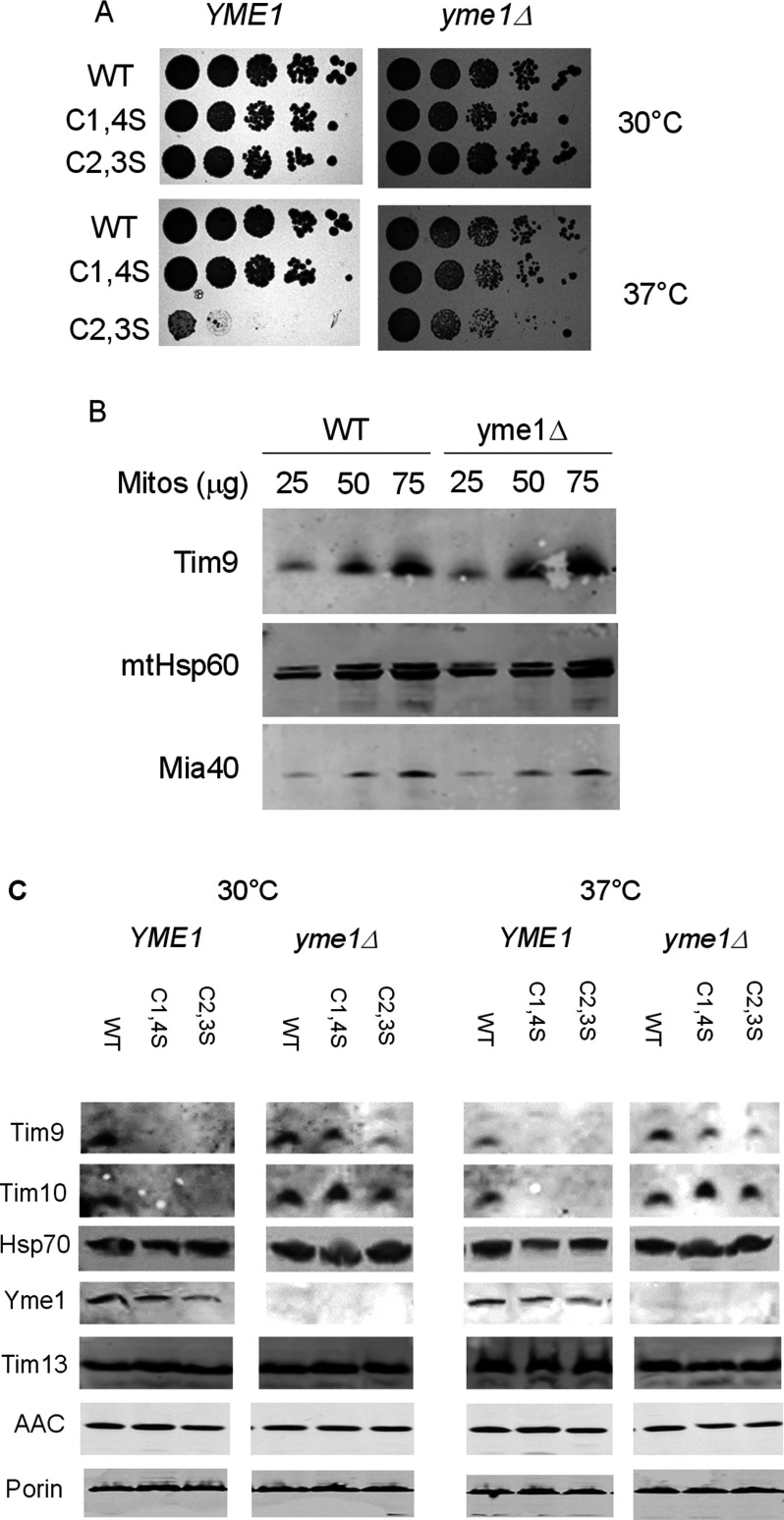
Deletion of *YME1* rescues *tim9C2,S* growth defect and Tim10 mitochondrial levels (**A**) Spot tests for growth of *tim9* mutants in the *yme1Δ* background on YPD media at 30°C and 37°C. (**B**). Western blot for levels of mitochondrial proteins in *YME1* and *yme1Δ* mitochondria. Mitochondria were lysed and proteins detected with the indicated antibodies (**C**). Western blots for levels of mitochondrial proteins in WT and *tim9* mitochondria in the presence or absence of Yme1. Isolated mitochondria were pre-incubated at either 30°C or 37°C for 15 min and then lysed. Fifty microgram was separated by SDS/PAGE and protein levels were detected using the indicated antibodies.

The reduced level of Tim9C2,3S protein led us to test whether absence of the IM AAA protease Yme1 might rescue the growth defect of the *tim9C2,3S* mutation. We deleted the *YME1* gene by PCR-based homologous recombination and replaced WT Tim9 with our mutant versions. We tested the growth of the strains at the above temperatures and found that the growth defect of the *tim9C2,3S* strain was almost totally suppressed by deletion of *YME1* (Figure 2A). This result suggests that the growth defect of the *tim9C2,3S* mutant is caused by protein degradation by the Yme1 protease and to further investigate this we examined whether *YME1* deletion caused a generalized increase in Tim9 levels. Firstly, we found that the levels of Tim9 in mitochondria purified from WT yeast were not significantly increased by deletion of *YME1* (Figure 2B).

Next and more specifically, we were interested in whether the suppression of the *tim9C2,3S* mutant growth defect could be rescued by restoration of Tim9 levels. We purified mitochondria from *TIM9* WT and mutant strains, either in WT or in *yme1Δ* background and analysed protein levels by Western blot, comparing the levels of Tim9 and Tim10 following incubation of mitochondria at 30°C and 37°C (Figure 2C). We found that levels of Tim9, and particularly Tim10, were increased in the *tim9C1,4S* mutant in the absence of Yme1. Surprisingly, we found that Tim9 levels in the temperature-sensitive *tim9C2,3S* mutant were only slightly enhanced at 37°C by deletion of the Yme1 protease (lane 9 compared with 12), but the levels of its partner protein Tim10 were significantly restored in the same mutant. Meanwhile, the levels of the non-essential Tim13 were unaffected by deletion of *YME1* and no effects were observed on the levels of small Tim clients AAC and Porin. These results suggest that, in purified mitochondria, the primary target for the Yme1 protease is Tim10 rather than Tim9 and thus that the rescue of cell growth in the *tim9C2,3S yme1Δ* mutant is a result of increased stability of Tim10.

**Figure 3:**
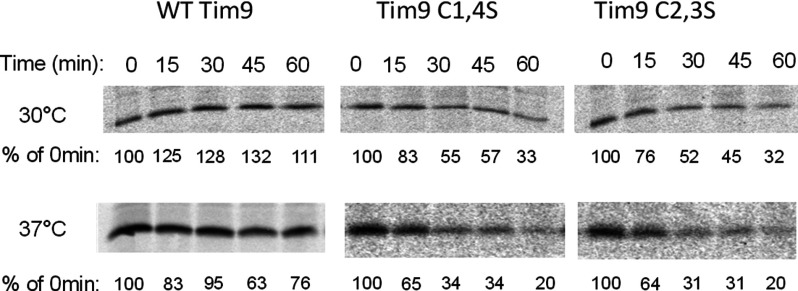
Pulse-chase analysis of Tim9 levels in WT and *tim9* cells Cells were grown at 30°C to early log phase, then pulse-labelled with ^35^S-methionine for 5 min. Following the addition of 20 mM cold methionine and 10 μg/ml cycloheximide, samples were incubated at 30°C or 37°C and then frozen in 10% TCA at the specified time points. Tim9 antibodies were used to immunoprecipitate Tim9 and labelled Tim9 was detected by autoradiography. Levels were quantified and expressed as a percentage of intensity at 0 min.

To further confirm this result, we tested small Tim protein stability *in vivo* by pulse-chase. Firstly, we examined Tim9 stability at 30°C and 37°C in WT and mutant strains. Following 5 min of labelling, translation was blocked and cells were chased with cold methionine. Chases were performed over a 1 h time course at either 30°C or 37°C and Tim9 was purified by IP. Results showed that WT Tim9 protein was stable over the course of the chase at both temperatures (Figure 3), whereas in agreement with our data from purified mitochondria (Figure 1D), both Tim9 mutants were unstable at 30°C and even more unstable at 37°C, dropping to ∼30% by 30 min.

**Figure 4:**
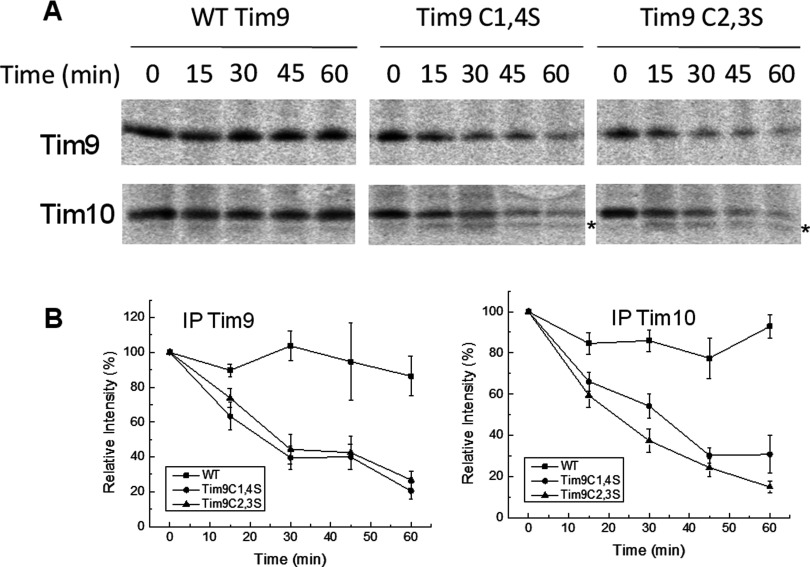
Pulse-chase analysis of Tim9 and Tim10 levels in WT and *tim9* cells (**A**) The same pulse-chases as in Figure 3, but all samples were chased at 37°C and then split and immunoprecipitated with Tim9 or Tim10 antibody as indicated. The Tim10 degradation product is indicated with an asterisk (*). (**B**) Quantification of results shown in (**A**) plotted as a percentage of the 0 min time point. Error bars represent the S.E.M., *n* = 3. Student's *t*tests (one-tailed, independent samples) showed no significant difference in the stability of the Tim9C1,4S and Tim9C2,3S mutants (*P*>0.3), but both mutants were significantly less than Tim9 WT (*P*<0.01). The levels of Tim10 in the *tim9C1,4S* and *tim9C2,3S* mutants were significantly less than in the WT strain (*P*<0.01).

Next, we tested whether Tim10 was also unstable across these time courses. Cells carrying WT, Tim9C1,4S or Tim9C2,3S plasmids pulse-labelled and chased as above and levels of both Tim9 and Tim10 were monitored at each time point by IP (Figure 4A). Instability of Tim9 in both mutant strains correlated with an equally severe instability of Tim10. Notably, in the *tim9C1,4S* and *tim9C2,3S* mutants, a faster migrating band (*∼9 kDa) was observed for Tim10 (most probably a degradation product) and was most prominent after 30 min in the *tim9C1,4S* mutant and after 15 min in the *tim9C2,3S* mutant. Results from three separate experiments are quantified and plotted in Figure 4(B), confirming both Tim9 and Tim10 have decreased levels in both of the *tim9* mutants.

**Figure 5:**
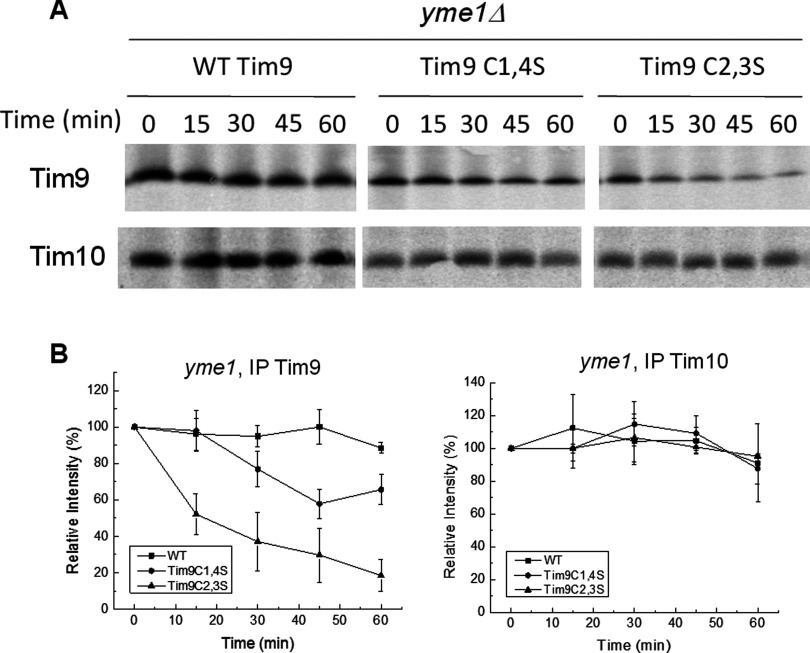
Deletion of *YME1* restores Tim10 levels, but not Tim9, in the *tim9C2,3S* mutant (**A**) The same pulse-chases as in Figure 4, but carried out in a *yme1Δ *background. **(B)** Quantification of results shown in (**A**) plotted as a percentage of the 0-min time point. Error bars represent S.E.M., *n*=3. Student's *t*tests showed deletion of *YME1* recovered the level of Tim9C1,4S so that it was significantly greater than Tim9C2,3S (*P*<0.01), whereas still significantly less than Tim9 WT (*P*<0.01). The levels of Tim10 in all three strains in the *yme1Δ *background, showed no significant difference between each other (*P*>0.3).

To investigate the role of Yme1 in the instability of both Tim9 and Tim10, we performed the same pulse-chase experiments with the WT or *tim9* mutants in the *yme1Δ* strain (Figure 5A). In the *yme1Δ *strain, Tim9C2,3S remained highly unstable, whereasTim9C1,4S levels were somewhat recovered. Contrastingly, in both these cases Tim10 levels in the *yme1Δ* strain were significantly higher than in the *YME1* strain, with no obvious decrease in Tim10 level over the time courses in not only the WT but also the *tim9* mutant strains (Figure 5A, lower panels). Furthermore, no ∼9 kDa Tim10 degradation product was observed in any sample purified from *yme1Δ* yeast, suggesting that the band observed under WT *YME1* background (Figure 4A, lower panels) was a product of Tim10 cleaved by Yme1. The results from three separate experiments were quantified and plotted (Figure 5B). Statistical analysis by Student's *t*test showed that Tim9 levels in the *yme1Δ* background remained significantly different between WT, Tim9C1,4S and Tim9C2,3S (*P*<0.01). Though deletion of *YME1* caused a partial recovery in the levels of the Tim9C1,4S mutant protein, there was no significant difference in Tim9C2,3S protein levels between the *YME1* WT and *yme1Δ* strains (*P* = 0.2). By contrast, Tim10 levels in *TIM9* cells and the two *tim9* mutants in a *yme1Δ* background showed no statistically significant differences (*P*>0.3), suggesting that Tim10 stability in these *tim9* mutants had been restored to the WT levels.

Thus, the rescue of the growth defect of the *tim9C2,3S* mutant by deletion of the Yme1 protease correlates not with a restoration of Tim9 levels, but with an increased level and stability of Tim10. Our results suggest that the Yme1 protease more specifically targets unassembled Tim10, rather than Tim9 mutant for degradation. We reasoned that in the presence of Yme1, an important function of Tim9 is to stabilize Tim10 in the hexameric Tim9–Tim10 complex. The resulting instability of Tim10 in the Tim9 mutant strains may then be due to poor interaction between these two proteins, causing Tim10 to exist primarily in monomeric form and thus be vulnerable to digestion by Yme1.

### Characterization of the cysteine mutants of Tim9 using purified proteins

**Figure 6:**
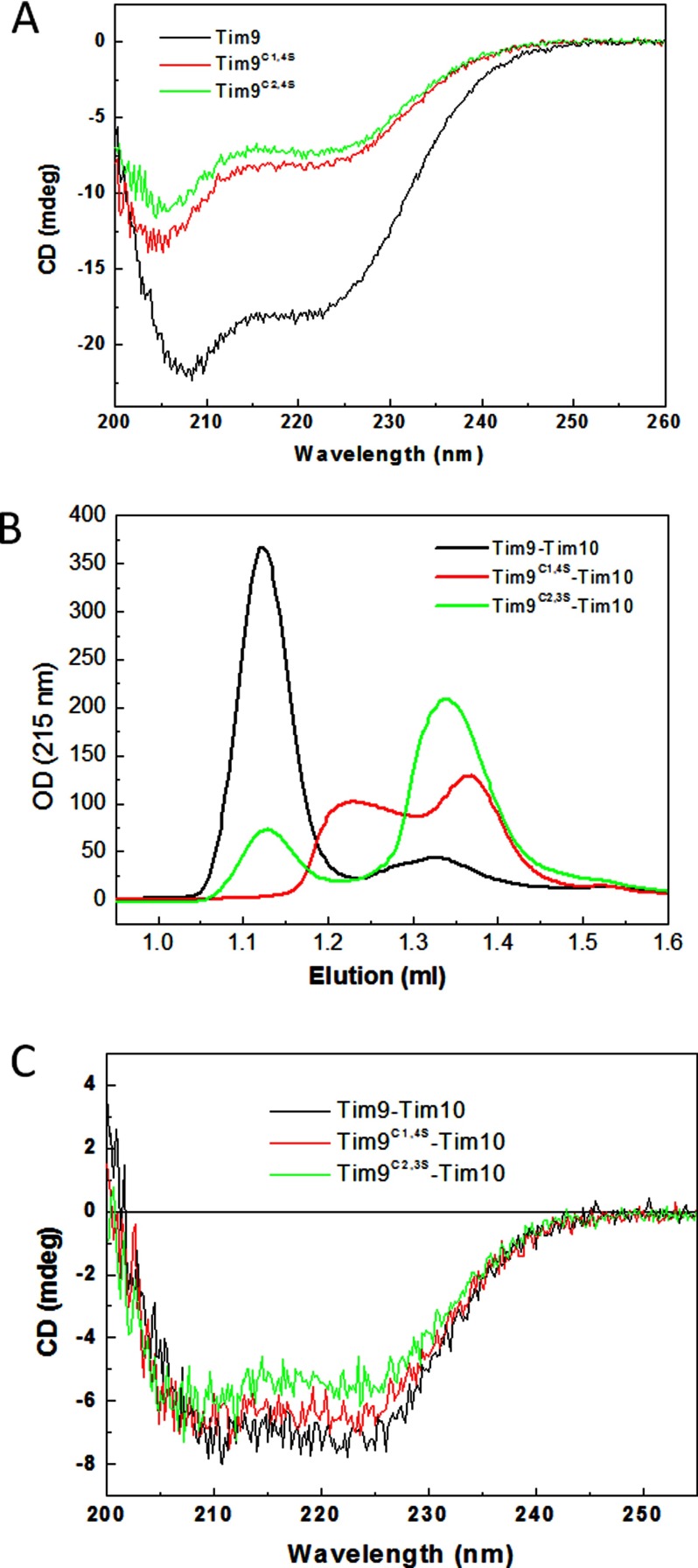
Mutant small Tim proteins form aberrant complexes *in vitro* (**A**) Far UV CD spectra of the WT and mutant Tim9 of (10 μM) at 25°C. (**B**) Gel filtration chromatography profiles of the WT and mutant Tim9 after incubation with the WT Tim10 at molar ratio of 1:1 in buffer AE. (**C**) Far UV CD spectra of the WT and mutant small Tim complexes (3 μM) at 5°C. All the complexes were pre-assembled and isolated using gel filtration chromatography.

To determine whether the *tim9* mutants result in unstable Tim10 due to inability to form the Tim9–Tim10 hexameric complex, two corresponding double cysteine mutant constructs were generated as GST fusions (see ’Experimental’). The recombinant mutant Tim9^C1,4S^ and Tim9^C2,3S^ were expressed and purified from *E coli* using the same method used for the WT protein [18] and the GST-tag was cleaved-off before final purification. Though both mutants could be successfully purified, their yields were much lower than that of the WT Tim9, indicating the mutants may be less stable. Overall folding of the mutants was studied using far-UV CD to see whether loss of either disulfide bond perturbed the secondary structure of Tim9. As shown in Figure 6(A), both mutants displayed a characteristic spectrum containing both α-helical and irregular structures as the WT protein, but their intensities were much lower than the WT Tim9. A typical CD spectrum of α-helix has two negative peaks at 222 and 208 nm, with a CD_222_/CD_208_ ratio of approximately 1.1 [39]. The ratio of CD_222_/CD_208_ was 0.83 for the WT, 0.70 for Tim9^C1,4S^ and 0.73 for Tim9^C2,3S^ respectively, confirming that both double cysteine mutants contained more unfolded structure and thus were less folded than the WT Tim9. This result is consistent with the result that the intramolecular disulfide bonds play a key role in stabilizing the folding of small Tim proteins [8,9].

Whether the Tim9 mutants are capable of forming a complex with the partner protein Tim10 was analysed using a FPLC gel filtration column chromatography (Superdex 75) after incubation of the WT Tim10 with the WT or mutant Tim9 at 1:1 molar ratio (Figure 6B). Whereas a fraction of Tim9^C2,3S^ was able to form the apparent same-sized complex with Tim10 as the WT Tim9, Tim9^C1,4S^ did not form the same-sized complex with Tim10. Instead, approximately 50% of the mutant proteins formed a small complex with an intermediate size between the hexameric Tim9–Tim10 complex and unassembled proteins. Moreover, the overall profile of the CD spectra of these three complexes were similar (Figure 6C), unlike that of the individual proteins (Figure 6A). The ratios of CD_222_/CD_208_ were increased to 1.12, 0.99 and 0.94 for the WT and the mutant complexes respectively. Together with the fact that the CD_222_/CD_208_ ratio for Tim10 is 0.8 [37], we conclude that protein folding was induced in both the WT and the mutant proteins upon complex formation.

### Tim9 mutant complexes are less stable than the WT complex

**Figure 7:**
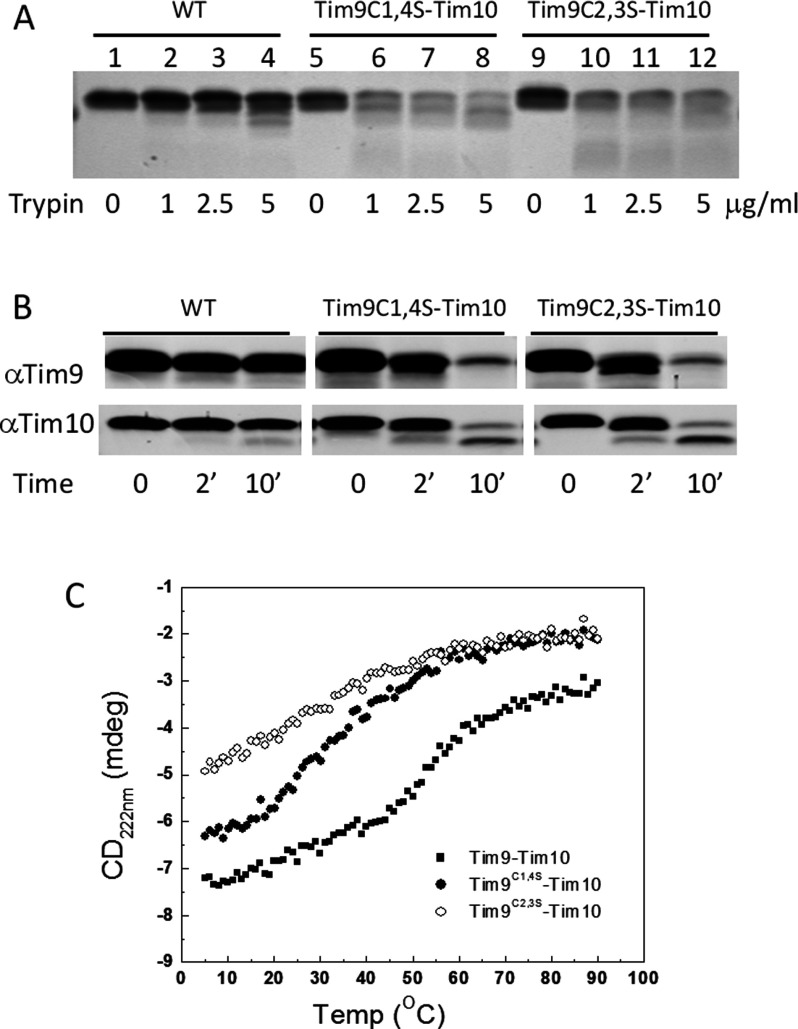
Mutant small Tim complexes are unstable (**A**) Trypsin digestion of pre-assembled WT and mutant small Tim complexes at RT for 30 min. The samples were analysed using 16% Tris/Tricine SDS/PAGE and Coomassie staining. (**B**) As in (**a**) but 2 μg/ml trypsin was used for the indicated times and the samples were analysed by Western blotting with antibodies against Tim9 and Tim10 respectively (**C**) Thermal denaturation of the pre-assembled WT and mutant small Tim complexes measured by CD at 222 nm.

To test whether Tim9 mutants were protected from protease degradation in the complexes, we incubated pre-assembled complexes with trypsin at various concentrations at RT for 30 min, after which the reactions were stopped by addition of trypsin inhibitor and analysed by SDS/PAGE (Figure 7A). The results showed that both mutant complexes were sensitive to trypsin digestion. The result was further confirmed by time course analyses of trypsin digestion (5 μM/ml) followed by western blotting using antibodies against Tim9 and Tim10 respectively (Figure 7B). For the WT complex, both Tim9 and Tim10 were protected against degradation over the time course. However, both Tim9 mutant complexes were sensitive to trypsin digestion, as the mutants failed to protect both themselves and WT Tim10 from degradation. We hypothesized that formation of the Tim9–Tim10 complex shields the small Tim proteins from degradation and the Tim9 mutants leave Tim10 vulnerable to degradation because the mutant complexes are unstable and/or more dynamic than the WT complex.

To gain more quantitative understanding about the effects of the Tim9 mutation on the stability of the complex, thermal denaturation of the complexes at the same protein concentration were studied by measuring CD intensity change at 222 nm, over 5°C–95°C (Figure 7C). For the WT complex, as shown previously [38], there is a gradual intensity decrease at temperatures below 40°C due to the presence of flexible tentacle-like N- and C-terminals in the complex; they undergo conformational change (unfolding) with temperature. The sharp intensity decrease between 40°C and 60°C resulted from the complex dissociation, with a melting temperature (*T*_m_) at approximately 52°C. A similar denaturation profile was obtained for Tim9^C1,4S^–Tim10 complex, but shifted towards low temperature and low intensity, with an apparent *T*_m_ of approximately 30°C. In the case of Tim9^C2,3S^–Tim10 complex, only one phase of intensity decrease at temperatures below 60°C was observed and the curve was further shifted towards low temperature and low intensity. The complex dissociation *T*_m_ cannot be determined but it seems to be below 20°C. It can be estimated that at 30°C whereas most of the WT proteins were in complex, approximately a half of Tim9^C1,4S^ and substantially less than half (∼25%) of Tim9^C2,3S^ molecules were forming a complex with WT Tim10. Thus, this result of our *in vitro* study correlates well with the *in vivo* results showing a temperature-sensitive growth phenotype for the *tim9C2,3S* mutant (Figure 1B).

## DISCUSSION

Early work on the biogenesis of the small Tim proteins focused on their import and the role of Mia40 in this process and concluded that oxidative folding, with formation of both pairs of disulfide bonds, is essential for the biogenesis of these proteins [9,15,40]. Oxidative folding of the cysteine-containing IMS proteins is thought to be required for both their stability and their retention in the IMS [16]. However, an earlier work [24] showed that mutation of any one of the four cysteine residues of Tim9 or Tim10 did not have any effect on cell viability and furthermore that instability of mutant Tim9 correlated with instability of WT Tim10 and that the levels of these proteins were restored by deletion of the i-AAA protease Yme1. Our results generally support the present work, as we observe that mutation of either disulfide bond of Tim9 results in instability of Tim10 and absence of Yme1 re-stabilizes Tim10. Furthermore, in the present study, we extend this finding to show that the temperature-sensitive growth defect observed due to mutation of the inner disulfide bond of Tim9 (Tim9C2,3S) can be rescued by deletion of Yme1 and that this strongly correlates with increased stability of Tim10 (despite continued instability of Tim9).

Although our findings are generally in agreement with the work of Baker et al. [24], some of our observations do differ. Firstly, our plate spot tests showed a stronger temperature-sensitive phenotype than [24], although they did see a strong growth defect when *tim9C2,3S* yeast was cultured in liquid medium. Secondly, we see a much stronger recovery of Tim10 levels than Tim9 from deletion of the Yme1 protease based on both Western blot and pulse-chase experiments at 37°C, whereas the previous work performed Western blot analysis for the steady-state levels of Tim9 and Tim10 in mitochondria and detected a recovery of both proteins under these conditions. However, we note that in solubilized mitochondria from *yme1Δ tim9C2,3S* cells, a significant build-up of monomeric Tim10, but not Tim9 was observed in [24]. This is in agreement with our conclusion that Yme1 predominantly acts to degrade unassembled Tim10, rather than (misfolded) Tim9. Such a difference may partially due to the fact that unassembled Tim10 is a monomer, whereas Tim9 forms homodimer *in vitro* [19]. The dimerization could induce a degree of folding and protection to Tim9 from degradation by proteases.

To complement the *in vivo* studies, we characterized the Tim9 mutants and their effects on Tim9–Tim10 complex formation using purified proteins *in vitro*. For both the Tim9 mutants we tested, strong defects in hexameric complex formation were observed, despite both mutants being able to support cell growth at permissive temperatures *in vivo*. Specifically, our data suggest that the Tim9 outer disulfide mutant (Tim9^C1,4S^) forms an intermediate-sized complex with Tim10 in a trimer and/or tetramer complex, with no stable hexameric complex formed (Figure 6B), yet does not show any growth defect *in vivo* (Figure 1B). This suggests that formation of the full Tim9–Tim10 hexamer might be dispensable for cell growth, as long as there are enough stable Tim9 and/or Tim10 present. In support of this conclusion, the Tim9^C2,3S^ mutant, which can form the 70 kDa hexameric complex with Tim10, but with a low affinity as evidenced by most of the proteins being unassembled under the same conditions (Figure 6B), has growth defects *in vivo*. It is also consistent with our observation that both Tim9 and Tim10 are present in a significantly decreased level in *tim9C2,3S* mutant yeast strain in a WT *YME1* background (Figure 2C). Moreover, our results from *in vitro* thermal stability studies of the WT and mutant complexes (Figure 7C) provided a good explanation for the *in vivo* temperature-sensitive growth phenotype *in vivo* (Figure 1B). The concept of Tim10 as the functional subunit of the small Tim proteins, whereas Tim9 plays a stabilizing role, has been previously suggested by [29], based on peptide spot assays. They showed that Tim10 alone displayed a similar binding pattern to client peptides as the Tim9–Tim10 complex and that Tim9 did not appear to bind to client. Our current study supports this hypothesis and further suggests that the Tim9–Tim10 hexameric complex may function purely or primarily as a stabilizer for Tim10 subunits. The stability of Tim10 depends not only on its oxidative folding, but also on its association with Tim9, which prevents its degradation by Yme1. Previous studies show that the oxidized but un-assembled Tim9 and Tim10 are folded in a molten-globule-like conformation that is sensitive to protease digestion [8]. Further folding is induced in the proteins upon complex formation; even for the largely unfolded cysteine mutants of Tim9 as demonstrated by CD analysis (Figure 7) [8]. This is consistent with and reinforces our current finding that Tim10 requires a protection by forming a stable complex with Tim9 to prevent from degradation by Yme1. Taken together, the results of this and previous studies suggest that (1) Tim10 rather than the Tim9–Tim10 complex is the functional form of these proteins and the primary function of Tim9 is to stabilize Tim10 from degradation by Yme1 via assembly with Tim10 [8,29]. (2) Formations of both disulfide bonds are not essential for the mitochondrial import and function of the small Tim proteins and formation of the inner disulfide bond in Tim9 is more important than the outer disulfide. Formation of the second (outer or inner) disulfide facilitates the assembly and stabilizes the hexameric Tim9–Tim10 complex.

A further quality control system for the IMS has been proposed to exist in the cytosol, where the ubiquitin proteasome has been shown to degrade un-imported IMS protein precursors [41]. Bragoszewski et al. [41] suggested that the degradation of IMS proteins by the cytosolic ubiquitin protease may be a continuous quality control mechanism, continuously removing a small percentage of precursor. The stabilizing activity of Tim9–Tim10 complex formation and the degradation activity of Yme1 may form a similar cycle in the IMS, with dissociation of the Tim9–Tim10 hexamer allowing a small proportion of Tim10 to be degraded and removed.

## Online data

Supplementary data
