# Supplementary material for: Mitochondrial Tim9 protects Tim10 from degradation by the protease Yme1
Source: Biosci Rep. 2015 May 19;35(3):e00193. doi: 10.1042/BSR20150038 (PMC4438305; doi:10.1042/BSR20150038)
Supplement: Supplementary data [file bsr035e193ntsadd.pdf]

Supplementary Table S1. Oligonucleotide primers used in this study.

**Primer name**	**Sequence**
9C1F	CTAATCTGGTAGAAAGAAGTTTCACAGACTGTGTC
9C1R’	GACACAGTCTGTGAAACTTCTTTCTACCAGATTAG
9C2F	GAAAGATGTTTCACAGACTCCGTCAATGACTTCACAACATC
9C2R	GATGTTGTGAAGTCATTGACGGAGTCTGTGAAACATCTTTC
9C3F	CAATAAGGAACAAACATCCATCATGAAGTGCTCAG
9C3R	CTGAGCACTTCATGATGGATGTTTGTTCCTTATTG
9C4F	CAAACATGCATCATGAAGTCCTCAGAAAAGTTCTTGAAGC
9C4R	GCTTCAAGAACTTTTCTGAGGACTTCATGATGCATGTTTG
9loxF	ACAGTATTTTACGGGGCACAAATCAAGAACAATAAGACAGCACTGTAAAGCCAC
9loxR	AATGGGCTACACCAGAGATACATAATTAGATATATATACGCCAGTACACCCCGC
Yme1F1	ATAATACATTGTGGATAGAACGAAAACAGAGACGTGATAGCGGAT
Yme1R1	GAGGTAGGTTCCTTCATACGTTTAACTTCTTAGAATAAAAGAATT
